# Thalidomide and its analogues in the treatment of Multiple Myeloma

**DOI:** 10.1186/2162-3619-1-27

**Published:** 2012-09-11

**Authors:** Tahir Latif, Nabeel Chauhan, Rashid Khan, Andrea Moran, Saad Z Usmani

**Affiliations:** 1 UC Cancer Institute, Division of Hematology-Oncology, University of Cincinnati School of Medicine, Cincinnati, OH, USA; 2 Myeloma Institute for Research & Therapy, University of Arkansas for Medical Sciences, 4301 W. Markham Street, Little Rock, AR, USA; 3 Carolyn & Neag Comprehensive Cancer Center, Lea’s Center for Hematologic Disorders, University of Connecticut Health Center, Farmington, CT, USA

**Keywords:** Multiple Myeloma, Thalidomide, Lenalidomide, Pomalidomide, Therapy

## Abstract

Multiple myeloma is an incurable malignant disorder of mature B-cells that predominantly affects the elderly. The immunomodulatory drug (IMiD) thalidomide and its newer analogs demonstrate increased antitumor activity, and have had a positive impact on the natural history of multiple myeloma. Recent advances in the clinical application of these agents and in our understanding of their mechanism of action, and toxicity have made safer and smarter use of these drugs possible. This review discusses the available information regarding mechanisms of action, toxicity and clinical results on thalidomide, lenalidomide and pomalidomide in the therapy of multiple myeloma.

## Introduction

**Figure 1:**
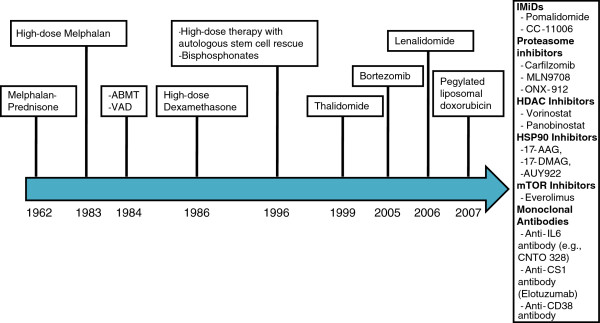
Timeline of advances in myeloma therapy.

Multiple myeloma is a mature B-cell neoplasm characterized by a monoclonal expansion of plasma cells in the bone marrow often accompanied by hemocytopenias, immunodeficiency, osteolytic lesions, hypercalcemia and renal failure. Myeloma accounts for about 10% of all hematological malignancies and 1% of all cancers [1]. In United States alone an estimated 20,520 new cases (11,400 men and 9120 women) will be diagnosed in 2011 and 10,610 people will die of myeloma [2]. Ever since the first reported case of ‘*mollities ossium’* described in 1844 [3], the disease has remained incurable, despite a better understanding of its pathogenesis and recent advancements in therapeutics. Prior to the advent of alkylating agents, the median survival for multiple myeloma was 1–1.5 years [4]. Following the introduction of L-phenylalanine mustard or melphalan in 1958 [5] and prednisone in 1962 [6], the combination of these two drugs remained the cornerstone of therapy for more than two decades, even though the complete remission rate remained less than 5% and the median survival with this treatment did not exceed 3 years [7]. Since the late 1990s, a number of new classes of drugs have been incorporated in the treatment of multiple myeloma and additional agents are under investigation (Figure 1). The present paper will review the clinical data for the use of thalidomide and its analogs, collectively referred as immunomodulatory drugs or IMiDs in multiple myeloma.

### The history and toxicity of immunomodulatory drugs (IMiDs)

**Figure 2:**
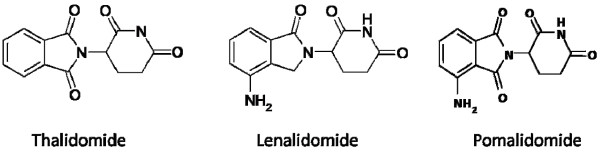
Molecular stutctures of the IMiD.

Thalidomide was first introduced as an oral sedative and anti-emetic in 1957, but was quickly abandoned due to its profound teratogenic effects. Almost four decades passed before studies demonstrated thalidomide to have anti-cancer properties, specifically showing significant *in vitro* anti-myeloma activity. It found its use as a novel anti-myeloma drug for relapsed and refractory disease in 1999 [8], later yielding impressive overall response rates of up to 50% when used in combination with dexamethasone and up to 65% when combined with corticosteroids and cyclophosphamide [9]. Thalidomide, though effective, was associated with dose-limiting toxicities including somnolence, constipation, neuropathy [10], and increased incidence of venothromboembolism (VTE), especially when combined with dexamethasone [11]. Hence, a new class of thalidomide derivatives called IMiDs was developed that, albeit structurally related (Figure 2, Table 1), had their unique set of anti-inflammatory, immunomodulatory, antiproliferative, antiangiogenic and toxicity profiles [12].

**Table 1:** Pharmacokinetics of the IMiDs

**Drug**	**Route of administration**	**Dosage**	**Metabolism/Excretion**	**Half-life**	**Dose adjustments**
Thalidomide	Oral	50-100 mg	Hepatic/Non-renal	4-9 hours	None at present
Lenalidomide (CC-5013)	Oral	15-25 mg	Renal (67% unchanged)	3.1-4.5 hours	Adjust for moderate to severe renal impairment
Pomalidomide (CC-4047)	Oral	1-5 mg	Renal (67% unchanged)	6.2-7.9 hours	None at present

Lenalidomide (CC 5013) was FDA approved for use in patients with relapsed and/or refractory multiple myeloma after successful clinical trials [13,14]. Lenalidomide has a distinct adverse effect profile compared to thalidomide. Notably, there is an increased risk of grade 3, or greater myelosuppression; however, the rates of sedation, constipation and neuropathy are all lower than thalidomide. The risk of VTE varies among studies, and is dependent on age, concurrent chemotherapy including dexamethasone, and the use of VTE prophylactic agents. Importantly, lenalidomide adversely affects adequate peripheral blood stem cell collection in older patients with therapy duration greater than 4 months [15,16]. More recently, several investigators have reported an alarmingly high rate of second malignancies, both myelodysplastic syndrome/acute myeloid leukemia and solid tumors in newly diagnosed patients with multiple myeloma receiving maintenance lenalidomide after autologous stem cell transplant (Table 2) [17-19]. However, additional data reported in 2011 Annual Meeting of American Society of Clinical Oncology did not show any increase in the rate of second malignancies in transplant ineligible and relapse or refractory patients undergoing lenalidomide therapy [20-22]. For example pooled data from MM-009/010 trials evaluating lenalidomide and dexamethasone versus dexamethasone/placebo and a phase II trial evaluating clarithromycin, dexamethasone and lenalidomide in 72 newly diagnosed multiple myeloma patients found similar frequency of second malignancies to background incidence and 2010 SEER data respectively for non-Multiple Myeloma individuals of similar age.

**Table 2:** Lenalidomide and incidence of second primary malignancies

**Study**	**Patient population**	**Treatment**	**Numbers**	**SPM**
MM-015	Elderly transplant ineligible patients	MP	153	(3%)
		MPR	152	(7%)
		MPR-R	150	(7%)
IFM 2005-02	Newly diagnosed post stem cell transplant	Placebo	302	11 (4%)
		Lenolidomide	306	26 (8%)
CALGB 100104	Newly diagnosed post stem cell transplant	Placebo	229	6 (2.6%)
		Lenolidomide	231	18 (7.8%)

Pomalidomide (CC-4047), another derivative of thalidomide has shown promising activity in phase I/II trials in relapsed and refractory multiple myeloma patients, including patients who have been treated with thalidomide, lenalidomide and the 26S proteasome inhibitor bortezomib (PS-341) [23-26]. Overall pomalidomide was well tolerated, with thromboembolic events the major non-hematological dose limiting toxicity, while neutropenia was the most common dose-limiting toxicity noted in initial phase 1 study. Peripheral neuropathy was reported by up to 30% of patients; however, the majority of these were reversible grade 1toxicity [23,24].

### Mechanisms of action

The mechanism of action of the IMiDs is not completely understood. They are thought to possess anti-proliferative, anti-angiogenic and immunomodulatory effects against myeloma cells. Lenalidomide and pomalidomide exert their anti-myeloma effect dually through direct down-regulation of key functions of the tumor cell, and indirectly by modulating interaction of myeloma cells with their microenvironment. They carry out their direct effects via modulation of cell adhesion and decreasing production of key pro-survival cytokines such as TNFalpha, IL-6, IL-8, and VEGF that favor tumor cell survival and proliferation, inhibition of apoptosis, and resistance to therapy [27].

Both lenalidomide and thalidomide inhibit TNFalpha production by increasing the degradation of TNFalpha mRNA and by increasing the activity of alpha-1-acid glycoproteins, which have intrinsic anti-TNFalpha activity [28]. In addition to the anti-angiogenic effect of thalidomide, IMiDs have further shown to directly induce apoptosis by triggering the activation of caspase-8, which in turn induces myeloma cell death via Fas-mediated pathways. Dexamethasone also activates caspase-9 pathway, hence forming the basis of its synergy with the IMiDs. Lenalidomide also utilizes mitochondrial pathways to release pro-apoptotic proteins such as cytochrome c and Smac [29]. Dexamethasone too, has been shown to release Smac, but not cytochrome c, in myeloma cells, hence providing further molecular basis for the additive effects of the two drugs, as seen in clinical trials.

IMiDs have shown to down-regulate NF-κB transcription activity *in vitro*, and in effect blunt out the survival advantage that myeloma cells employ in tumorigenesis. Dexamethasone has also been shown to down regulate NF-κB activity, and coupled with lenalidomide nearly abrogates NF-κB activity in myeloma cells [30]. The net effect is inhibition of caspase inhibitors, induction of caspase-8 activity, and promotion of Fas- and TRAIL/Apo2L- induced apoptosis. IMiDs also block the stimulatory effect of insulin like growth-factor-1 on NF-κB activity and potentiate the activity of dexamethasone and proteasome inhibitor therapy.

Furthering their immune modulatory role, the IMiDs also affects other cellular components in the bone marrow. In preclinical studies they have demonstrated activation of T and NK cells [31-33]. Lenalidomide induces T-cell proliferation, mainly by co-stimulation of CD28 that in turn leads to increased transcription and production of IL-2 and IFN-gamma. It is in fact many times more potent than its predecessor. As a result of increased T-cell derived IL-2 and IFN-gamma, and lenalidomide and pomalidomide have been shown to augment CD 56+ NK cell numbers and function [34].

In addition to its diverse immunomodulatory effects, lenalidomide, although a weaker inhibitor of angiogenesis than thalidomide, has a well-documented anti-angiogenic profile. Instead of directly inhibiting endothelial cell proliferation like its predecessor, lenalidomide in fact inhibits the secretion of vascular endothelial growth factor (VEGF) and basic fibroblast growth factor (bFGF), from both tumor and stromal cells [35]. Lenalidomide inhibits Akt phosphorylation, hence interrupting the phosphatidylinositol 3-kinase (PI3K)/Akt signaling pathway, known to be integral in tumor cell survival and proliferation. Inhibition of this pathway, downstream of VEGF signaling may explain in part the drug’s anti-proliferative and anti-angiogenic effects. Lenalidomide also inhibits VEGF-induced PI3K-Akt pathway signaling, which is known to regulate adherens junction formation. A strong inhibitory effect of lenalidomide on hypoxia-induced endothelial cell formation of cords and hypoxia-inducible factors (HIF-1) alpha expression, the main mediator of hypoxia-mediated effects and a key driver of angiogenesis and metastasis, has also been found [36]. Pomalidomide and lenalidomide also demonstrated their potential in epigenetic modulation of p21 WAF-1 expression, thereby possibly being efficacious in p53 mutated tumors [37].

Recent landmark discovery of cereblon (CRBN), an essential protein to induce thalidomide teratogenicity in zebrafish and chicken embryos, led to several presentations at 2011 American society of Hematology (ASH) meeting demonstrating the critical role of CRBN in anti-proliferative response to IMiDs [38,39]. Majority of myeloma cell lines and patients resistant to IMiDs showed very low levels of CRBN [40]. In addition, bone marrow CRBN mRNA expression was also predictive of response to lenalidomide and dexamethasone in small study of 44 patients [41].

### Clinical data

#### Thalidomide

Thalidomide-based chemotherapy combinations are currently considered front-line regimens in both transplant eligible and transplant ineligible patients. Initial phase I data was first described in patients with relapsed and refractory multiple myeloma in 1999 by Singhal et al [9]. A number of subsequent trials utilizing thalidomide combinations have demonstrated overall response rates of 46%-67% for relapsed and refractory disease (summarized in Table 3).

**Table 3:** Clinical trials-thalidomide (Relapsed/refractory disease)

**Author**	**Regimen**	**Type of clinical trial**	**No. of pts**	**Best responses (>PR)**	**CR, nCR, VGPR**	**PFS/TTP/OS (months)**	**Reference**
Barlogie et al	T	Phase II	169	30%	CR:2%	2 yr EPS:20% 2 yr OS;48%	Blood 2001
Palumbo et al	TD	Phase II	62(FR), 58(SR)	56% (FR), 46% (SR)	Not reported	PPS:17(FR),11(SR) OS:60%(FR,3-yr),19(SR)	Hematol J 2004
Dimpopoulus et al	TD	Phase II	44	55%	>VGPR:30%	Median survival 12.6	Annal Oncol 2001
Moehler et al	DT-PACE	Phase II	50	65%	CR:4%	PPS:15 1 yr OS:63%	Bllod 2001
Terpos E et al	VMDT	Phase II	62	66%	CR:13% VGPR:27%	TTP: 9.7	Leukemia 2008
Palumbo et al	VMDT	Phase II	30%	67%	VGPR:43%	PPS (1 yr):61% OS (1 yr):84%	Blood 2007

Four different phase III trials have evaluated the use of thalidomide in combination with melphalan/prednisone (MP) in comparison with MP in transplant in-eligible patients (Table 4). One of the four trials added thalidomide maintenance until relapse [42]. The MPT regimen demonstrated superior overall responses and complete responses compared to MP in two of these trials. Although Palumbo et al. also showed improved overall responses in comparison to MP, but the initial difference in overall survival was not seen with longer follow-up, questioning the justification of thalidomide maintenance. One of these trials evaluated the optimal treatment strategy in the elderly MM patients by comparing MP, MPT and high-dose therapy/autologous stem cell transplant [43]. This trial showed that MPT had better overall survival than the other two arms. Based on these trials, MPT is considered a front-line regimen for transplant ineligible and elderly MM patients.

**Table 4:** Clinical trials-thalidomide (Transplant Ineligible)

**Author**	**Regimen(s)**	**Type of clinical trial**	**No. of pts**	**Best responses (>PR)**	**CR, nCR, VGPR**	**PFS/TTP/OS (months)**	**Reference**
Palumbo et al	MPT+T maintenance vs. MP	Phase III	255	76% vs 47.6%	CR:15.5% vs. 2.2%	OS:45 vs. 47.6 PPS:21.8 vs. 14.5	Blood 2008
Facon et al	MPT vs MP vs. HDT/ASCT	Phase III	427	89% vs 37% vs 83%	CR:13% vs. 2% vs. 18% VGPR:47% vs. 7% vs. 43%	OS:51.6 vs. 33.2 vs. 38.3	Lancet 2007
Hulin et al	MPT vs MP	Phase III	229	69% vs 39%	CR:7% vs 1% VGPR:22% vs. 7%	OS:45.3 vs. 27.7 PPS:24.1 vs. 19.0	Blood (ASH Annual Meeting) 2007; 110:75 (abstr)
Ludwig et al	MPT vs. MP	Phase III	231	68% vs 51%	CR:14% vs. 7% nCR:17% vs.8% VGPR:17% vs. 14%	OS:45 vs. 58 EPS:25 vs. 43	Blood (ASH Annual Meeting) 2007; 110:529 (abstr)
Palumbo et al	MPT	Phase I/II	53	81%	CR:23.8% VGPR:47.6%	1 yr EPS:92% 1 yr OS:100%	J Clin Oncol 2007

Thalidomide-based combinations have also been evaluated as induction regimen for transplant-eligible myeloma patients (Table 5). It has been observed that combination of 2 novel agents results in better depth of response, as seen in combination of thalidomide with bortezomib and dexamethasone (VTD). The VTD is often the induction regimen of choice outside of clinical trials, with overall response rates of 93%. Currently three-novel drug and four-novel drug regimens are being evaluated in clinical trials for safety and efficacy. This strategy certainly holds promise judging by the encouraging results of the Total Therapy 3 data (utilizing VTD-PACE), demonstrating that combining all active anti-myeloma agents upfront results in overall responses approaching 100%, and 87% of the complete responders remain disease free four years from initial therapy [44].

**Table 5:** Clinical trials-thalidomide (Transplant Eligible)

**Author**	**Regimen(s)**	**Type of clinical trial**	**No. of pts**	**Best responses (>PR)**	**CR, nCR, VGPR**	**PFS/TTP/OS (months)**	**Reference**
Rajkumar et al	TD vs. D	Phase III	470	63% VS. 46%	CR+VGPR:44% vs. 16%	TTP:22.6 vs. 6.4	J Clin Oncol 2008
Lockhorst et al	TAD vs. VAD	Phase III	402	72% VS. 54%	NR	NR	Haematologica 2008
Cavo et al	VTD vs. TD	Phase III	187	93% VS. 79%	NR	NR	ASH 2007
Barlogie et al	VTD-PACE	Phase II	303	99%	nCR:83% (2 yr) CR: 56% (2 yr)	EFS:84% (2 yr) OS: 86% (2 yr)	B J Hematol 2007

Thalidomide had also been evaluated as maintenance therapy in several trials. As an example, the recently published MRC Myeloma IX trial showed statistically significant improvement in progression free survival, 23 vs. 15 months; log-rank *P* <0.001, in patients receiving low dose (50 to 100 mg) thalidomide maintenance as compared to placebo. There was no significant difference in median overall survival *P* = 0.40. However, a meta-analysis performed by same authors including additional data from other published studies evaluating thalidomide maintenance showed significant late survival benefit *P* <0.001 [45].

#### Lenalidomide

In recent years, lenalidomide has become the lead IMiD in myeloma clinical investigation due to its superior safety profile. Two phase II trials have looked at the efficacy of lenalidomide and dexamethasone in comparison to dexamethasone alone, showing superior overall responses, complete response and overall survival (Table 6). This combination is also superior to dexamethasone alone in newly diagnosed patients (Table 7). The efficacy of this combination was marred by the unacceptably high rates of venous thromboembolism (26%). An ECOG phase III trial comparing low-dose dexamethasone to previously utilized high-dose dexamethasone, found not only reduction in VTE rates (12%) in the low-dose group, but also an improved overall survival at the first interim analysis resulting in suspension of the study [46]. The high-dose group was subsequently switched to the low dose dexamethasone regimen. Niesvizky et al. have shown in a phase II study that addition of clarithromycin to lenalidomide and dexamethasone (BiRD) results in a higher overall response rate [47].

**Table 6:** Clinical trials-lenalidomide (Relapsed/refractory disease)

**Author**	**Regimen(s)**	**Type of clinical trial**	**No. of pts**	**Best responses (>PR)**	**CR, nCR, VGPR**	**PFS/TTP/OS (months)**	**Reference**
Dimopoulus et al	LD	Phase III	351	60.2% vs. 24%	CR+nCR:15.9% vs. 3.4%	TTP:11.7 vs. 4.7 OS:Not reached vs. 20.7	N Engl J Med 2007
Weber et al	LD	Phase IIII	353	61% vs. 19.9%	CR:14.4 vs. 0.6%	TTP:11.7 vs. 4.7 OS:29.6 vs. 20.2	N. Engl J Med 2007
Palumbo et al	MPL-L	Phase III	459	77% vs. 49%	CR:18% vs. 5%	Not reached	ASH 2009 ASCO 2010

**Table 7:** Clinical trials-lenalidomide (Newly diagnosed)

**Author**	**Regimen(s)**	**Type of clinical trial**	**No. of pts**	**Best responses (>PR)**	**CR, nCR, VGPR**	**PFS/TTP/OS**	**Reference**
Rajkumar et al	Ld vs. LD	Phase III	445	79% vs. 69%	CR/nCR:18% vs. 14% VGPR:33% vs. 26%	1-year OS:96% vs. 87%	Lancet Oncol 2010
Zonder et al	LD vs. D	Phase III	133	85% vs. 51%	NR	Not reported	ASH 2007
Richardson et al	LVD	Phase I/II	35 (Phase II)	100%	VGPR:69% CR + nCR:54%	Not reached at 19.3 months (median)	ASH 2009
Jakubowiak et al	LV-PLD-D	Phase I/II	26 (Phase II)	96%	VGPR:67% CR + nCR:33%	Not achieved at 6 months (median)	ASH 2009
Kumar et al	LCD vs. VCD vs. LCVD	Phase II	117	90% vs. 87% vs. 94%	VGPR:33% vs. 35% vs. 42% CR:12% vs. 6% vs. 15%	Not reported	ASH 2009
Niesvizky et al	Clarithromycin + LD	Phase II	72	90.3%	VGPR:16.7% CR + sCR:38.9%	Actuarial EFS (2yr): 85.2% (transplant), 75.2% (non-transplant)	Blood 2008
Palumbo et al	MPL	Phase I/II	54	81%	VGPR:47.6% CR:23.8%	EFS (1 yr):92% OS (1 yr):100%	JCO 2007

With the successful results of combining MP with thalidomide and then with bortezomib in the elderly myeloma population, the addition of lenalidomide to MP was assessed by Palumbo et al. in a phase I/II trial [48]. The study demonstrated overall response of 81% with majority of these patients achieving a very good partial response (VGPR) or better. The results have led to initiation of an ECOG phase III trial comparing MPT with MPL as the first line therapy for newly diagnosed transplant ineligible myeloma patients.

A number of phase I and II trials are also presently exploring the combination of lenalidomide with other novel anti-myeloma agents. Promising results have been observed with the combination of lenalidomide, dexamethasone and bortezomib in both newly diagnosed and relapsed/refractory patients. Updated analysis of phase I/II trial reported at ASH 2011 showed an overall response rate of 98%, including 71% VGPR and 36% CR/nCR for 68 newly diagnosed patients, response rate was 100% for 52 patients treated at maximum planned dose [49]. Similarly impressive results were seen in relapse/refractory patients with median time to progression of almost 10 months and response rate of 78% including 25% complete response [50].

Based on these observations, an ongoing MMRC sponsored phase I/II trial evaluating the combination of lenalidomide, dexamethasone, bortezomib and liposomal doxorubicin as first line therapy in newly diagnosed patients showed greater than 96% partial response with 30% complete response in 57 evaluable patients [51]. A Mayo Clinic lead randomized phase II trial is comparing the combination of bortezomib, cyclophosphamide, lenalidomide and dexamethasone with either bortezomib or lenalidomide with cyclophosphamide and dexamethasone [52]. Although initial reports of these studies demonstrate unprecedented overall response rates for the four-drug combinations. It remains to be seen whether the depth of response may lead to similar success, and potentially, cures such as observed in aggressive lymphomas.

Lenalidomide had also shown promising activity as maintenance therapy after autologous stem cell transplant, e.g. McCarthy et al. [17] showed median time to progression of 46 months vs. 27 months (P < 0.001) and Attal et al. [18] showed median progression-free survival of 41 months, vs. 23 months (P < 0.001) in the lenalidomide and placebo groups respectively [19,20]. Lenalidomide was also evaluated in transplant ineligible patients as part of initial and maintenance therapy. In this trial patients received melphalan, prednisone, lenalidomide (MPR) as nine 4-week cycles of MPR followed by lenalidomide maintenance therapy until relapse or disease progression or MPR or MP without maintenance therapy. The median progression-free survival was significantly longer with MPR-R (31 months) than with MPR (14 months; P *<* 0.001) or MP (13 months; P *<* 0.001).

#### Pomalidomide

Pomalidomide is the newest IMiD that is undergoing clinical investigation (Table 8). Initial response rates for relapsed and refractory disease exceed expectation, even in the patients who have lenalidomide refractory disease (PR = 32%). In an ongoing phase II study, patients who had failed both lenalidomide and bortezomib were given pomalidomide and dexamethasone. To date the response rates with this regimen exceed 50% with an OS of 86% after 6 months [53]. Treatment benefit with pomalidomide and low dose dexamethasone was also confirmed by another Phase II study in patients with multiple myeloma despite prior use of lenalidomide [54]. In a phase II trial presented at the 2011 American Society of Hematology meeting, 46 relapse and refractory patients treated with pomalidomide, dexamethasone, and clarithromycin (ClaPD), showed overall response rate of 60% with 27% showing very good partial remission [55]. Another study evaluating pomalidomide, cyclophosphamide, prednisone (PCP) in 41 patients with relapse refractory multiple myeloma including lenalidomide refractory patients show 59% partial response including 2 patients achieving complete response [56]. More research is needed to establish the optimal treatment strategy and role of pomalidomide in patients with relapsed and refractory myeloma.

**Table 8:** Clinical trials-pomalidomide

**Author**	**Regimen(s)**	**Type of disease/ clinical trial**	**No. of pts**	**Best responses (>PR)**	**CR, nCR, VGPR**	**PFS/TTP/OS (months)**	**References**
Lacy M et al	Pd	Relapsed & refractory disease/Phase I	60	63%	CR:5% VGPR:28% PR:30%	PFS:11.6	J Clin Oncol 2009
Schey et al	P	Relapsed & refractory disease/Phase I	24	54%	CR:17%VGPR:13% PR:25%	Not reported	J Clin Oncol 2004
Streetly et al	P	Relapsed & refractory disease/Phase I	20	50%	CR:10% VGPR:30% PR:10%	PFS:10.5 OS:33	Br J Haematol 2008
Lacy M et al	Pd	Lenalinomide & bortezomib refractory disease/Phase I	34	50%	VGPR:3% PR:29% MR:18%	Not reported	ASCO 2010
Richardson P et al	Pd	Lenalinomide refractory disease/Phase I	32	52%	PR:28%	Not reported	ASH 2009

## Conclusion

After over two decades with limited treatment options, numerous new regimens incorporating novel agents are now available for both transplant eligible and transplant ineligible patients with multiple myeloma. While response rates, adverse reactions and the optimal dosing strategy vary among the various IMiDs, there is increasing data to suggest that these novel agents and regimens are altering the natural history of the disease, improving the quality of life and longevity of patients with myeloma. Although teratogenicity is a significant concern and careful monitoring and precautions are required when using these agents, recent discoveries on the possible mechanism of thalidomide teratogenicity will hopefully lead to development of safer IMiDs [38]. Immunomodulatory drugs are a promising class of therapy for patients with multiple myeloma, both as single agents and in combination with conventional chemotherapy.

## Competing interests

SZU has served as a consultant to Celgene, Millennium, and Onyx. SZU has received research funding from Celgene and Onyx, and speaking honoraria from Celgene and Millennium. TL has received speaking honoraria from Milllennium.

## Authors’ contributions

All authors participated in preparation of the manuscript. All authors have read and approved the final manuscript.
